# Clinical and Histopathological Factors Associated with the Tumoral Expression of TGF-*β*1, MED15, CD16, and CD57 in Oral Squamous Cell Carcinoma

**DOI:** 10.1155/2022/3145117

**Published:** 2022-10-27

**Authors:** Maryam Elahi, Vahid Rakhshan

**Affiliations:** ^1^Department of Pathology, Alborz University of Medical Sciences, Karaj, Iran; ^2^Department of Dental Anatomy, Dental School, Azad University of Medical Sciences, Tehran, Iran

## Abstract

**Introduction:**

Factors associated with the expression of oral squamous cell carcinoma (OSCC) biomarkers “CD16, CD57, TGF-*β*1, and MED15” are not assessed, except in few controversial studies of some of these biomarkers. This study aimed to highlight factors that can correlate with tumoral overexpression of these biomarkers.

**Methods:**

In this genetically-matched case-control study, biomarker expressions in *all* available OSCC tissues and their adjacent normal tissues at the National Tumor Center (*n* = 384 (4 biomarkers × (48 cancers + 48 controls))) were measured using qRT-PCR. Factors associated with tumoral overexpression of CD16, CD57, TGF-*β*1, and MED15 (compared to the benign control) were evaluated, using log-level multiple linear regressions and Spearman (*α* = 0.05).

**Results:**

Tumoral CD16 upregulation was observed in younger patients (*β* = −0.284, *P*=0.040) and cigarette smokers (*β* = 0.397, *P*=0.005). Tumoral CD57 was upregulated in males (*β* = 0.341, *P*=0.008), smokers (*β* = 0.401, *P*=0.002), and cases without vascular invasion (*β* = −0.242, *P*=0.042). Tumoral TGF-*β*1 was elevated in smokers (*β* = 0.452, *P*=0.001) and smaller tumors (*β* = −0.322, *P*=0.045). Tumoral MED15 was overexpressed in smokers (*β* = 0.295, *P*=0.036) and cases lacking perineural invasion (*β* = −0.394, *P*=0.007).

**Conclusion:**

As the most consistent finding, smoking might be positively associated with tumoral overexpression of all biomarkers. Tumoral increase in CD57 might be positively associated with metastasis while being negatively correlated with vascular and lymphatic invasion. Tumor size might be negatively associated with tumoral TGF-*β*1 expression.

## 1. Introduction

As the most frequent oral cancer with a poor diagnosis, oral squamous cell carcinoma deserves a great deal of research attention [1, 2]. It is of clinical and scientific interest to assess factors that can contribute to prognostic tumor biomarkers. Some prognostic biomarkers are not assessed adequately (or at all) in terms of their associated factors. These include MED15, transforming growth factor beta 1 (TGF-*β*1), CD16, and CD57 [2, 3].

TGF-*β*1 is a cytokine with contradictory properties [4–6]. It can both suppress and induce tumorigenesis depending on the situation even in one tumor type [3–6]. Studies on factors associated with its tumoral overexpression are a few and controversial [6].

CD16 is an IgG Fc receptor on the surface of inflammatory cells [3, 7]. It plays a key role in antibody-dependent cellular cytotoxicity which is responsible for defense against tumor cells [3, 7]. In this regard, any factors associated with tumoral expression of CD16 have not been assessed in SCC or any other cancers.

CD57 is a surface antigen usually expressed in monocytes, T-lymphocytes, and NK cells which are major attackers of cancer cells and inducers of antigen-specific response [3, 7–10]. Therefore, changes in CD57 levels are expected to be observed in cancer tissues. Although certain factors have been identified to accompany the overall increase of CD57 expression in healthy subjects, the associations between prognostic factors and tumoral expression of this marker (in comparison to its expression in healthy tissues) have not been assessed except in a few studies [11–13].

MED15 is a main regulator of various signaling pathways including TGF-*β* signaling [14, 15]. It has been recently linked to cancer [3, 14, 15] but no studies exist on factors contributing to its tumoral overexpression.

Besides the abovementioned shortcomings and controversies, the previous studies had adopted less accurate measuring methods such as immunohistochemistry. Moreover, many of them had adopted the less accurate bivariable statistical analyses which can confound the results when the predictors are interconnected.

Due to abovementioned major gaps in the literature, this study was conducted. It aimed to highlight, for the first time, the independent clinical and histopathological predictors of tumoral overexpression of the above four biomarkers in OSCC, measured using the accurate method of real-time quantitative polymerase chain reaction (qRT-PCR). For conducting this study, we used the National Tumoral Bank data which had been used in our previous study as well [3]. Our previous paper was about the prognostic and diagnostic values of these 4 biomarkers [3]. Nevertheless, this new article is about histoclinical factors determining the tumoral overexpression of these biomarkers. Thus, it is an independent, new paper, and without any smallest overlap between the predictors and results or other parts of this paper and our previous paper—which was about something else (except the parts regarding genetic assessments) [3].

## 2. Materials and Methods

This was a genetically-matched case-control study. The sample consisted of all the 48 OSCC tumors available from the National Tumor Bank, as well as their adjacent normal tissues treated as control specimens. The sample size was decided as all the available tissues (*n* = 384 biomarker data points = 4 markers × (48 OSCC cancer tissues + 48 control tissues)). Originally 49 tumors and 49 healthy tissues were collected. However, after reassessing the tumors, it was found that one of them was basal cell carcinoma and therefore, was excluded along with its healthy tissue. Diagnosis was done based on histopathological examinations at two intervals by a minimum of two pathologists. No patients had undergone radiotherapy or chemotherapy before surgery. The ones excluded were patients with any inflammatory diseases or any other tumor. Histopathological and clinical records of patients were evaluated and collected as independent variables. Linear measurements were estimated twice by two pathologists. The protocol and its ethics were approved by the research committee of the university (approved as theses 1395-113 and 1395-118) [3].

RNA extraction and real-time quantitative polymerase chain reaction (qRT-PCR) were done thrice for every 96 normal and cancerous tissues. The same tissue samples (which had been used for histopathological assessments) were used for RNA extraction and qRT-PCR as well. Primer sequences were synthesized for CD16 (left: GTGGGTGTTCAAGGAGGAAG, right: CTGCCTTTGCCATTCTGTAA), CD57 (left: GAACTTGTCACCCTCAACGA, right: CTTCTTGCCCTCATTCACC), TGF-*β*1 (left: AGCTGTACATTGACTTCCGC, right: GTCCAGGCTCCAAATGTAGG), and MED15 (left: AGAACTTCAGTGTCCCCTCA, right: GTACTTCGACAGCTGCTTCA). Extracted RNAs were normalized to 1 *μ*g. Afterwards, single-strand cDNA was produced by reverse-transcribing the RNA by a thermo kit (Thermo Fisher Scientific, Waltham, Massachusetts, USA). A Nano-Drop Technologies (ND-2000) device was used to analyze the purity and quantity of the extracted RNA. Quantitative polymerase chain reaction was performed according to the protocol of Bioneer RT-PCR thermal cycler using SYBR green/ROX (Takara, Japan) real-time PCR master mix. After commencing the amplification protocol, the delta threshold cycle value (ΔCt) for each sample (Ct_(Housekeeping)_-Ct_(Target)_) was calculated for relative expression of the studied genes to the housekeeping gene (*β*-actin). The next item calculated was “Delta Delta” cycle value (ΔΔCt) as the+log_2_-fold-change; it was calculated from the difference between the ΔCt of the tumoral tissue and the ΔCt of its healthy tissue (i.e., tumor ΔCt-benign ΔCt). Positive ΔΔCt points increase in tumoral expression of a biomarker compared to benign tissue expressions [3].

### 2.1. Statistical Analysis

Bivariate associations between variables were estimated using chi-square, a Fisher, an unpaired *t*-test, and a Spearman correlation coefficient. A log-level stepwise backward-selection multiple linear regression was used to evaluate the factors associated with the ΔΔCt of each of the biomarkers. The log-level multiple linear regressions were performed on ΔΔCt values of the four markers (as the dependent variables) using pairwise deletion criterion. Some variables were excluded because of having very few variations (i.e., alcohol consumption which had only 1 positive case). Some others were removed and replaced with composite scores (i.e., pathological T, pathological N, and clinical metastasis, which were replaced with the stage). The independent variables were sex (dichotomized, reference was “male”), age (continuous), tumor size (continuous), tumor volume (continuous), tumor depth (continuous), histology grade of tumor (ordinal), necrosis (dichotomized), lymphatic invasion (dichotomized), vascular invasion (dichotomized), perineural invasion (dichotomized), nodal extension (dichotomized), stage (ordinal), and smoking (dichotomized). In the regression analysis for each of the 4 genes, the independent variables were assessed for bivariate associations, multicollinearity, significance, the number of missing data, and the number of significant results in the backward-optimized model. In the case of inflated variances, one of the two correlated independent variables would be removed from the model, and the model would be compared in terms of the model fit and significance with the other versions (when the other variables were removed). The model with a reasonably low degree of variance inflation factor (VIF) would be optimized using backward-selection method. For stepping method criteria, 0.05 and 0.1 probabilities of *F* were used as entry and removal criteria, respectively. The software in use was SPSS 25 (IBM, Armonk, NY, USA). The level of significance was set at 0.05.

## 3. Results

**Figure 1:**
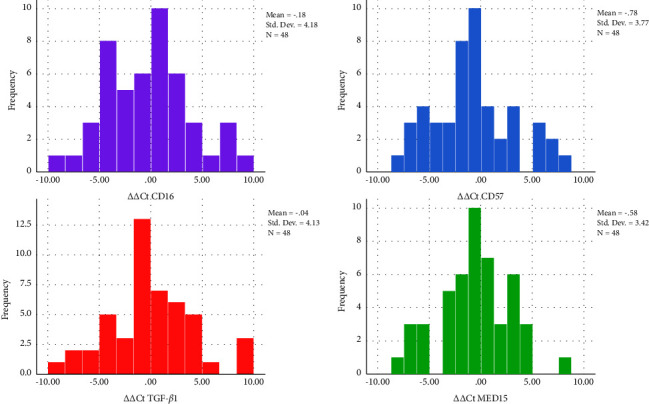
Histograms of ΔΔCt values along with descriptive statistics.

There were 29 men and 19 women. Histopathological properties of one of the patients were not available. Their age was 63.8 ± 15.3 years (range: 23.4–90.4). Males' age was 64.0 ± 13.8 years (range: 26.0–86.4). Females' age was 63.8 ± 17.8 years (range: 63.6–90.4). There was no significant difference between ages of the sexes (unpaired *t*-test, *P*=0.925). Average tumor sizes in men and women were 47.86 ± 22.09 and 46.05 ± 31.12 mm, respectively, (unpaired *t*-test, *P*=0.817). Mean tumor volumes in men and women were 40.86 ± 59.48 and 85.56 ± 201.38 ml, respectively, (unpaired *t*-test, *P*=0.273). Average tumor sizes in men and women were 47.86 ± 22.09 and 46.05 ± 31.12 mm, respectively, (unpaired *t*-test, *P*=0.817). Average depths of invasion in men and women were 15.63 ± 10.14 and 21.82 ± 19.68 mm, respectively, (unpaired *t*-test, *P*=0.273). The histological grades I, II, and III were seen in 28, 16, and 3 patients, respectively. Necrosis was present in 10 patients. Lymphatic invasion was observed in 10 patients. Vascular invasion existed in 9 patients. Perineural invasion was positive in 18 patients. Extracapsular nodal extension was seen in 3 patients. Stages 1 to 4 were observed in 4, 6, 11, and 26 patients, respectively. Except smoking that was more prevalent in men (as no women were smokers, *P*=0.033, Fisher's exact test), all other categorical variables were similarly distributed between males and females (chi-square or Fisher's exact tests, *P* > 0.1).  Figure 1 shows descriptive statistics and histograms of ΔΔCt values.

### 3.1. Factors Associated with Gene Expressions

There were significant moderate positive correlations among tumoral gene expression and also between smoking and tumoral expression of the three markers (Table 1).

#### 3.1.1. Multivariable Analysis of CD16 Predictors

The first model (Akaike Information Criterion (AIC) = 136.596, adjusted *R*-square = 0.115, *F* = 1.485, *P*=0.179) was optimized using a backward-selection procedure (i.e., the 9th model, AIC = 125.714, adjusted *R*-square = 0.203, *F* = 3.873, *P*=0.009, Table 2). Younger ages and smoking cigarettes were positively associated with increased CD16 expression in the tumor compared to its adjacent tissue.

Since the variable TNM stage was composed of the variables “pathological T, pathological N, and clinical metastasis,” they were reanalyzed separately in a new single model replacing the variable stage. None of the three variables became significant in any of the models (*P* > 0.1) and the results pertaining to the rest of the predictors were similar to those of the model with the variable stage instead of the 3 variables “pathological T, pathological N, and clinical metastasis”.

#### 3.1.2. Multivariable Analysis of CD57 Predictors

The first CD57 model (AIC = 113.160, adjusted *R*-square = 0.338, *F* = 3.084, *P*=0.006) and the model optimized using the backward-selection method (the 9th model, AIC = 102.018, adjusted *R*-square = 0.404, *F* = 11.167, *P* < 0.001) are presented in Table 3. Male gender, smoking cigarettes, and a lack of vascular invasion were positively associated with tumoral CD57 overexpression.

Since vascular invasion was highly correlated with lymphatic invasion, the regression was redone with lymphatic invasion instead of vascular invasion, in order to prevent multicollinearity. The optimized model (adjusted *R*-square = 0.396, *F* = 10.825, *P* < 0.001) consisted of the variables male sex (beta = 0.396, *P*=0.003), smoking (beta = 0.344, *P*=0.010), and lymphatic invasion (beta = −0.235, *P*=0.059).

The variables “pathological T, pathological N, and clinical metastasis” replaced stage and analyzed separately in a new model. The optimized model (adjusted *R*-square = 0.436, *F* = 7.962, *P* < 0.001) identified the variables clinical metastasis (beta = 0.281, *P*=0.046), male sex (beta = 0.306, *P*=0.015), smoking (beta = 0.418, *P*=0.001), and vascular invasion (beta = −0.236, *P*=0.043) as significant predictors.

#### 3.1.3. Multivariable Analysis of TGF-*β*1

The first TGF-*β*1 model (AIC = 131.402, adjusted *R*-square = 0.183, *F* = 1.914, *P*=0.073) and the optimized model (the 9th model, AIC = 122.466, adjusted *R*-square = 0.228, *F* = 5.441, *P*=0.003) are demonstrated in Table 4. Tumoral TGF-*β*1 was overexpressed in smokers and smaller tumors.

#### 3.1.4. Multivariable Analysis of MED15

The first model (AIC = 119.879, adjusted *R*-square = 0.071, *F* = 1.311, *P*=0.261) was optimized using a backward-selection procedure (i.e., the 7th model, AIC = 108.549, adjusted *R*-square = 0.198, *F* = 3.228, *P*=0.015, Table 5). Perineural invasion and cigarette smoking were negatively and positively associated with tumoral MED15 increase, respectively.

## 4. Discussion

Since studies on predictors of these biomarkers in OSCC or SCC were not available, we were limited to discussing our findings using the few studies on other tumor types. Besides, since there was no study on the factors associated with two of these biomarkers in any tumors, we had to discuss more general aspects of the matter. The clinical impact and application of this study are in determining the risk factors that can contribute to the overexpression of these tumor biomarkers, which themselves are indicators of the prognosis [3]. In the present study, CD16 upregulation in OSCC tumors (relative to its expression in adjacent benign tissues) was associated with none of the other independent variables except (negatively with) aging, and (positively with) smoking cigarettes. There were no other studies directly examining the cancerous expression of CD16 compared to controls (in any cancers), as a function of aging. Hence, we are limited to discussing more general aspects of this finding. It is not known why tumoral cells might express CD16 genes less than healthy tissues. In noncancerous individuals, a major source of CD16 expression is leukocytes such as natural killer cells, lymphocytes, and monocytes. Aging can affect the number, phenotypes, and functions of such cells. Aging might increase the CD-mature NK cells especially CD56−16+ and CD57+ ones [16, 17] (without overall cytotoxicity improvements); this possibly happens to compensate for impaired cytotoxic activity of individual NK cells that occurred because of senescence and inefficient signal transduction [16–18]. It is suggested that aging might not affect NK cell activation mediated by CD16 [16, 17]. However, according to some studies, CD16 expression might reduce in neutrophils of the elderly [19]. This might lower the phagocytic activity and immune competence [19]. We could not find studies on this particular matter (i.e., the effect of aging on CD16 expression in tumoral tissues in SCC or any other cancers) to discuss it further. It is anticipated that an older age might be associated with increased cancer risk, due to the accumulation of the carcinogenic effects over time as well as immune senescence in the elderly [18, 20].

Of all other independent variables, only three were associated with CD57 expression, two (smoking cigarettes and being a male) were independently associated with higher CD57 expressions in the tumor compared to the adjacent tissue. On the other hand, relative CD57 downregulation was found to be associated positively with the presence of vascular invasions. Some authors have reported a higher expression of CD57 in men compared to women [21] but we could not find studies on the relative expression of this marker in tumoral cells.

Our findings showed an increase in tumoral CD16 and CD57 expressions (compared to their expressions in benign tissues) in smokers. Effects of cigarette smoking on the CD16 expression have been controversial. As stated above, it might activate CD16 monocytes and increase their adhesion to the endothelium [22]. On the other hand, it has been linked to reduced CD16 NK cell counts [23, 24]. As well another study has found elevated granulocyte cell counts and CD16^+^–CD56^+^ cell counts [25]. Some studies found positive significant associations between smoking and the CD57 expression [26, 27] although some others did not report such associations [28]. Smoking might increase the cytotoxic T cells while having paradoxical effects on NK cells [29, 30]. These studies were concerned with the overall increase of these markers in smokers and not about their tumoral upregulation related to its expression in benign tissues. Our results might be indicative of an enhanced immunity in the tumor as a function of amplified inflammation in the tumor caused by smoking, or perhaps a weakened immune function in the benign tissue caused by possible mechanisms such as negative effects of smoking on vascularization [31]; it should be noted, however, that both smoking effects and inflammatory reactions are much more complicated to be explained straightforwardly. These deductions need future studies, and because of the lack of any relevant studies in this regard, further discussion was not possible.

Higher CD57 expressions in the tumor compared to the adjacent tissue were independently associated with being a male. Few authors have reported a higher expression of CD57 in men compared to women [21] but we could not find studies on the relative expression of this marker in tumoral cells (any tumor types) compared to normal cells.

In the current study, prognostic factors such as histological grade, tumor size or its thickness, and nodal involvement were not associated with tumoral overexpression of CD16 or CD57. In contrast to our findings, some of the few other available studies found some associations. Feng et al. [32] investigated the bivariable associations between CD14^+^–CD16^+^ monocytes with potential prognostic factors and showed negative relationships between this subset with tumor size and TNM staging [32]. According to Ishigami et al. [33], increased NK cell infiltrations in gastric cancer might be associated with a lower positivity of lymph node metastasis and lymphatic invasion [33]. Nevertheless, Akagi and Baba [34] did not observe associations between low and high percentages of CD57+ T cells and the variables “stage, lymph node metastasis, lymphatic invasion, vascular invasion, and tumor depth” in gastric cancer [34]. Fraga et al. [10] evaluated bivariate associations between CD57 expression with age, clinical staging, T parameter, N parameter, grade, and anatomic site and found merely the *N* parameter to be associated with tumoral CD57 increase [10]. Fang et al. [35] as well assessed bivariate relationships between tumoral CD57 infiltration with the variables age, gender, smoking, drinking, differentiation, *T* and *N* stages, and clinical stage in OSCC; the only significant associations existed between CD57 and *N* and clinical stage. The latter two studies were in contrast to our results, according to which the only significant association existed between CD57 and clinical metastasis of TNM. On the other hand, Lopes et al. [36] evaluated the link between the expression of CD57 with the factors “age, metastasis to regional lymph nodes, clinical stage, and histopathological grade”; they reported CD57 to be associated with none of them. The controversy might be attributed to the use of bivariate statistics, dichotomizing the variables, and different sample types (in terms of the tumor types, tumor progressions, and tumor sizes).

The prognostic factors independently associated with tumoral CD57 expression in this sample were clinical metastasis (as the only significant part of TNM staging), vascular invasion, and to a lesser degree lymphatic invasion. The present findings implied that the elevated tumoral levels of CD57 might be associated with the lack of vascular/lymphatic invasion as well as increased clinical metastasis. In addition, the vascular invasion was marginally associated with tumoral CD16 downregulation. CD57 is a marker of NK maturation [7–10] and therefore is expected to be positively associated with the prognosis. In prostate cancer, CD57 loss might be associated with tumor size and dedifferentiation [11]. Nevertheless, our findings indicated reduced relative expression of CD57 in cancers with poorer prognoses (implied by vascular invasion), which was in contrast to Nasir et al. [12] who reported increased CD57 cells in follicular carcinoma—which had vascular invasion—compared to follicular adenoma (which does not have vascular invasion). Khan et al. [13] as well observed a similar CD57 increase in papillary thyroid carcinoma compared to benign tissues. However, instead of comparing cancers with and without vascular invasion, they compared cancerous and noncancerous tissues. This methodological difference as well as differences in types and properties of tumors, sample, or statistical analyses might partly explain the dispute. Furthermore, several earlier studies had used bivariable statistics [10, 35, 36], while when assessing the role between prognostic factors and CD57/CD16 expression, we controlled for other prognostic factors (e.g., stage, grade, or size) using multivariable analyses; in this case, the negative effect found between the markers and vascular invasion was independent of those major prognostic factors. In addition, although tumoral CD57 expression can be used as a prognostic factor, it follows a heterogeneous pattern [11], making it difficult to derive straightforward linear correlations. Moreover, CD57 also increases in the normal tissue. Iida et al. [37] showed an increase in CD57 T-cells in peripheral blood of OSCC patients in line with increases in clinical stages, suggesting that such elevations are of prognostic value. Our results can be justified in light of an inefficient immune response in severer malignancies (which could also accompany vascular invasion) that led to a negative association with both mortality and vascular invasion.

Among all other independent factors in this study, tumoral TGF-*β*1 overexpression was only associated (positively) with smoking cigarettes. This finding was contrasting some studies suggesting that cigarette smoking might enhance tumorigenicity through attenuating TGF-*β*-mediated growth inhibition and apoptosis [38]. On the other hand, another study reported that nicotine might indirectly increase TGF-*β*1 expression [39]. Again, the role of TGF-*β*1 is much more complicated than simply expecting the same correlation in all situations. The lack of associations between TGF-*β*1 and age was in line with some studies [7, 40] and contrast to some others showing slight decreases by aging [6]. Also, some authors had found a positive role between higher TGF-*β*1 levels and being female [6] or tumor grade [6, 40]. Differences might be due to sample properties (cancer types and demographics) and methodologies.

A slight negative association was observed between tumoral MED15 expression and perineural invasion; cigarette smoking was positively associated with its expression as well. Although the link between cigarette smoking and MED15 might be somehow anticipated (as cigarettes contain numerous carcinogens), the negative association with perineural invasion could not be easily justified and needs future research. There was no previous study in these matters in any cancers or in noncancerous individuals to compare our results with.

### 4.1. Limitations and Advantages

This study was limited by some factors. Firstly, there was no previous study on the histoclinical predictors of some of these biomarkers and there were only a few studies regarding some others. This made comparing our results with the literature difficult and at points, impossible. The sample size was not based on power calculations. However, this sample consisted of all the available specimens in the National Tumor Bank. Moreover, the qPCR method is more accurate than other methods such as subjective IHC scorings; thus, given its very high precision and considerable expenditures of the assessments of these four biomarkers, the sample size of 96 genetically-matched tissues can be regarded as large. Earlier studies on a *single* biomarker had used less accurate methods on sample sizes of around 45 patients [10, 36, 37]. To our knowledge, this was the only qPCR study on these markers. Some may argue that alcohol must have been investigated in our research; we did want to investigate it, but of the 48 patients, only 1 reported the use of alcohol. Therefore, due to the lack of any variations in the sample, it was not possible to include this item. As an advantage, the number of the potential associated factors tested was larger than many of the other few studies. Moreover, we used multivariable statistical analyses which were absent in many of the previous few studies.

## 5. Conclusions

Smoking was positively associated with the tumoral overexpression of CD16, CD57, TGF-*β*1, and MED15. Younger ages were associated with tumoral CD16 upregulation, while the male sex was associated with tumoral CD57 overexpression. Vascular invasion vas associated with CD57 downregulation. Small tumor diameters were associated with tumoral TGF-*β*1 elevation. Perineural invasion was associated with tumoral MED15 downregulation.

## Figures and Tables

**Table 1:** The correlation matrix among variables, calculated using the Spearman coefficient.

		Sex: male	Age	Tumor size	Tumor volume	Tumor depth	Grade	Necrosis	Lymph invasion	Vascular invasion	Perineural invasion	Nodal extension	Stage	Smoking
Sex: male	Rho		−0.035	0.165	−0.013	−0.112	0.109	0.216	0.110	−0.040	0.025	0.038	0.182	0.334
	*P*		0.811	0.266	0.932	0.459	0.466	0.144	0.460	0.790	0.869	0.801	0.220	0.020

Age at diagnosis	Rho	−0.035		0.166	0.147	0.062	−0.249	−0.023	0.157	0.215	0.123	−0.212	−0.038	−0.019
	*P*	0.811		0.266	0.325	0.681	0.092	0.878	0.292	0.146	0.412	0.153	0.799	0.897

Tumor size (mm)	Rho	0.165	0.166		0.775	0.359	−0.014	0.123	0.376	0.200	0.311	−0.042	0.349	−0.111
	*P*	0.266	0.266		<0.001	0.014	0.928	0.409	0.009	0.177	0.033	0.780	0.016	0.459

Tumor volume (ml)	Rho	−0.013	0.147	0.775		0.653	−0.170	0.077	0.173	0.116	0.387	−0.083	0.305	−0.234
	*P*	0.932	0.325	<0.001		<0.001	0.252	0.608	0.246	0.439	0.007	0.577	0.037	0.114

Depth of invasion (mm)	Rho	−0.112	0.062	0.359	0.653		0.033	0.110	0.155	0.114	0.224	0.028	0.091	−0.167
	*P*	0.459	0.681	0.014	<0.001		0.826	0.467	0.302	0.451	0.134	0.852	0.546	0.267

Histology grade	Rho	0.109	−0.249	−0.014	−0.170	0.033		0.108	0.108	0.012	−0.110	0.348	0.336	0.109
	*P*	0.466	0.092	0.928	0.252	0.826		0.468	0.468	0.939	0.462	0.016	0.021	0.464

Necrosis presence	Rho	0.216	−0.023	0.123	0.077	0.110	0.108		0.238	0.011	−0.089	0.077	−0.119	0.075
	*P*	0.144	0.878	0.409	0.608	0.467	0.468		0.107	0.940	0.553	0.607	0.426	0.618

Lymphatic invasion	Rho	0.110	0.157	0.376	0.173	0.155	0.108	0.238		0.672	0.125	0.077	0.053	−0.217
	*P*	0.460	0.292	0.009	0.246	0.302	0.468	0.107		<0.001	0.402	0.607	0.723	0.142

Vascular invasion	Rho	−0.040	0.215	0.200	0.116	0.114	0.012	0.011	0.672		0.284	−0.127	0.090	−0.052
	*P*	0.790	0.146	0.177	0.439	0.451	0.939	0.940	<0.001		0.053	0.395	0.545	0.730

Perineural invasion	Rho	0.025	0.123	0.311	0.387	0.224	−0.110	−0.089	0.125	0.284		−0.027	0.164	−0.084
	*P*	0.869	0.412	0.033	0.007	0.134	0.462	0.553	0.402	0.053		0.859	0.270	0.576

Extracapsular nodal extension	Rho	0.038	−0.212	−0.042	−0.083	0.028	0.348	0.077	0.077	−0.127	−0.027		0.224	−0.109
	*P*	0.801	0.153	0.780	0.577	0.852	0.016	0.607	0.607	0.395	0.859		0.130	0.465

Stage	Rho	0.182	−0.038	0.349	0.305	0.091	0.336	−0.119	0.053	0.090	0.164	0.224		−0.151
	*P*	0.220	0.799	0.016	0.037	0.546	0.021	0.426	0.723	0.545	0.270	0.130		0.310

Smoking	Rho	0.334	−0.019	−0.111	−0.234	−0.167	0.109	0.075	−0.217	−0.052	−0.084	−0.109	−0.151	
	*P*	0.020	0.897	0.459	0.114	0.267	0.464	0.618	0.142	0.730	0.576	0.465	0.310	

ΔΔCt CD16 (log_2_-fold-change)	Rho	0.192	−0.250	−0.179	−0.013	0.053	0.133	0.119	−0.199	−0.255	−0.207	0.077	−0.169	0.322
	*P*	0.191	0.087	0.229	0.933	0.728	0.373	0.426	0.179	0.083	0.164	0.607	0.256	0.026

ΔΔCt CD57 (log_2_-fold-change)	Rho	0.469	−0.222	−0.206	−0.175	−0.105	0.110	0.103	−0.276	−0.271	−0.216	0.000	−0.030	0.475
	*P*	0.001	0.129	0.164	0.240	0.487	0.464	0.489	0.060	0.065	0.144	1.000	0.839	0.001

ΔΔCt TGF-*β*1 (log_2_-fold-change)	Rho	0.151	−0.090	−0.207	−0.063	0.048	0.156	0.073	−0.230	−0.146	−0.181	0.010	−0.009	0.409
	*P*	0.307	0.543	0.164	0.674	0.751	0.294	0.627	0.120	0.329	0.224	0.949	0.954	0.004

ΔΔCt MED15 (log_2_-fold-change)	Rho	0.029	−0.164	−0.031	0.004	0.026	0.015	0.084	−0.069	−0.191	−0.365	−0.038	−0.075	0.254
	*P*	0.844	0.266	0.838	0.979	0.865	0.923	0.573	0.645	0.198	0.012	0.797	0.614	0.082

**Table 2:** Factors associated with the ΔΔCt CD16, calculated using the backward-selection stepwise log-level multiple linear regression.

Models and parameters		Unstandardized coefficients		Beta	*P*	95% CI		VIF
		B	SE			Low	Up	
1	(Constant)	3.467	3.680		0.353	−4.020	10.953	
	Sex: male	1.517	1.500	0.180	0.319	−1.535	4.569	1.602
	Age at diagnosis	−0.060	0.043	−0.219	0.170	−0.146	0.027	1.246
	Tumor size (mm)	−0.027	0.032	−0.164	0.414	−0.092	0.039	2.000
	Depth of invasion (mm)	0.082	0.049	0.292	0.107	−0.018	0.182	1.576
	Histology grade	0.324	1.114	0.048	0.773	−1.943	2.592	1.394
	Necrosis presence	0.473	1.573	0.047	0.766	−2.727	3.672	1.234
	Lymphatic invasion	0.294	2.426	0.029	0.904	−4.642	5.230	2.937
	Vascular invasion	−1.408	2.377	−0.134	0.558	−6.243	3.427	2.605
	Perineural invasion	−0.945	1.380	−0.111	0.498	−3.754	1.863	1.342
	Extracapsular nodal extension	0.818	2.784	0.048	0.771	−4.846	6.482	1.380
	Stage	−0.484	0.775	−0.115	0.537	−2.062	1.093	1.713
	Smoking	3.696	2.007	0.316	0.075	−0.387	7.780	1.494

8	(Constant)	4.038	2.528		0.118	−1.071	9.147	
	Age at diagnosis	−0.067	0.037	−0.248	0.079	−0.143	0.008	1.072
	Tumor size (mm)	−0.031	0.026	−0.190	0.239	−0.083	0.021	1.442
	Depth of invasion (mm)	0.066	0.044	0.235	0.146	−0.024	0.155	1.432
	Vascular invasion	−1.671	1.441	−0.159	0.253	−4.584	1.242	1.074
	Smoking	4.555	1.569	0.389	0.006	1.385	7.726	1.023

9	(Constant)	4.455	2.513		0.084	−0.620	9.529	
	Age at diagnosis	−0.077	0.036	−0.284	0.040	−0.151	−0.004	1.015
	Tumor size (mm)	−0.033	0.026	−0.204	0.207	−0.085	0.019	1.434
	Depth of invasion (mm)	0.065	0.044	0.234	0.149	−0.024	0.155	1.432
	Smoking	4.646	1.573	0.397	0.005	1.468	7.823	1.021

B, regression coefficient; SE, standard error; beta, standard regression coefficient; CI, confidence interval; VIF, variance inflation factor.

**Table 3:** Factors associated with the ΔΔCt CD57, calculated using the backward-selection stepwise log-level multiple linear regression.

Models and parameters		Unstandardized coefficients		Beta	*P*	95% CI		VIF
		B	SE			Low	Up	
1	(Constant)	0.344	2.829		0.904	−5.405	6.094	
	Sex: male	2.772	1.116	0.364	0.018	0.505	5.040	1.456
	Age at diagnosis	−0.042	0.033	−0.171	0.206	−0.108	0.024	1.198
	Tumor size (mm)	−0.021	0.024	−0.143	0.384	−0.069	0.027	1.785
	Depth of invasion (mm)	0.029	0.038	0.117	0.447	−0.048	0.107	1.573
	Histology grade	−0.308	0.853	−0.051	0.720	−2.041	1.425	1.340
	Necrosis presence	0.080	1.208	0.009	0.947	−2.375	2.536	1.196
	Vascular invasion	−1.622	1.249	−0.171	0.203	−4.161	0.917	1.182
	Perineural invasion	−0.770	1.053	−0.100	0.470	−2.910	1.370	1.282
	Extracapsular nodal extension	0.056	2.111	0.004	0.979	−4.234	4.346	1.303
	Stage	0.226	0.584	0.059	0.702	−0.962	1.414	1.598
	Smoking	4.317	1.437	0.409	0.005	1.396	7.237	1.258

7	(Constant)	0.544	2.004		0.787	−3.506	4.595	
	Sex: male	2.669	0.925	0.350	0.006	0.799	4.539	1.133
	Age at diagnosis	−0.041	0.029	−0.166	0.169	−0.099	0.018	1.072
	Tumor size (mm)	−0.013	0.017	−0.088	0.452	−0.047	0.021	1.038
	Vascular invasion	−1.811	1.120	−0.191	0.114	−4.075	0.453	1.075
	Smoking	4.185	1.288	0.396	0.002	1.583	6.788	1.143

8	(Constant)	0.098	1.906		0.959	−3.752	3.947	
	Sex: male	2.618	0.918	0.343	0.007	0.764	4.472	1.127
	Age at diagnosis	−0.043	0.029	−0.174	0.145	−0.101	0.015	1.063
	Vascular invasion	−1.892	1.109	−0.200	0.096	−4.132	0.348	1.066
	Smoking	4.293	1.273	0.407	0.002	1.722	6.865	1.129

9	(Constant)	−2.532	0.719		0.001	−3.983	−1.080	
	Sex: male	2.601	0.931	0.341	0.008	0.723	4.480	1.127
	Vascular invasion	−2.291	1.092	−0.242	0.042	−4.494	−0.088	1.003
	Smoking	4.232	1.291	0.401	0.002	1.627	6.837	1.128

B, regression coefficient; SE, standard error; beta, standard regression coefficient; CI, confidence interval; VIF, variance inflation factor.

**Table 4:** Factors associated with the ΔΔCt TGF-*β*1, computed using the backward-selection stepwise log-level multiple linear regression.

Models and parameters		Unstandardized coefficients		Beta	*P*	95% CI		VIF
		B	SE			Low	Up	
1	(Constant)	0.902	3.450		0.795	−6.109	7.912	
	Sex: male	0.123	1.361	0.015	0.928	−2.642	2.889	1.456
	Age at diagnosis	−0.045	0.040	−0.168	0.263	−0.126	0.036	1.198
	Tumor size (mm)	−0.056	0.029	−0.348	0.062	−0.114	0.003	1.785
	Depth of invasion (mm)	0.075	0.047	0.271	0.118	−0.020	0.170	1.573
	Histology grade	0.043	1.040	0.006	0.967	−2.070	2.156	1.340
	Necrosis presence	0.878	1.473	0.088	0.555	−2.116	3.871	1.196
	Vascular invasion	−1.269	1.523	−0.122	0.411	−4.365	1.827	1.182
	Perineural invasion	−1.046	1.284	−0.124	0.421	−3.656	1.563	1.282
	Extracapsular nodal extension	−1.620	2.574	−0.097	0.533	−6.851	3.611	1.303
	Stage	0.883	0.713	0.211	0.224	−0.565	2.331	1.598
	Smoking	5.231	1.752	0.451	0.005	1.670	8.792	1.258

7	(Constant)	3.125	2.419		0.204	−1.763	8.013	
	Age at diagnosis	−0.045	0.035	−0.167	0.210	−0.116	0.026	1.031
	Tumor size (mm)	−0.040	0.026	−0.252	0.123	−0.092	0.011	1.538
	Depth of invasion (mm)	0.074	0.043	0.269	0.090	−0.012	0.161	1.435
	Perineural invasion	−1.315	1.170	−0.156	0.268	−3.680	1.051	1.164
	Smoking	5.218	1.513	0.450	0.001	2.161	8.274	1.024

8	(Constant)	3.325	2.420		0.177	−1.562	8.212	
	Age at diagnosis	−0.050	0.035	−0.185	0.162	−0.121	0.021	1.015
	Tumor size (mm)	−0.048	0.025	−0.299	0.061	−0.098	0.002	1.434
	Depth of invasion (mm)	0.072	0.043	0.260	0.101	−0.015	0.158	1.432
	Smoking	5.310	1.515	0.458	0.001	2.250	8.370	1.021

9	(Constant)	0.310	1.181		0.795	−2.074	2.693	
	Tumor size (mm)	−0.052	0.025	−0.322	0.045	−0.102	−0.001	1.417
	Depth of invasion (mm)	0.073	0.043	0.263	0.101	−0.015	0.160	1.431
	Smoking	5.235	1.533	0.452	0.001	2.142	8.328	1.019

B, regression coefficient; SE, standard error; beta, standard regression coefficient; CI, confidence interval; VIF, variance inflation factor.

**Table 5:** Factors associated with the ΔΔCt MED15, calculated using the backward-selection stepwise log-level multiple linear regression.

Models and parameters		Unstandardized coefficients		Beta	*P*	95% CI		VIF
		B	SE			Low	Up	
1	(Constant)	2.384	3.044		0.439	−3.801	8.570	
	Sex: male	−0.455	1.200	−0.066	0.707	−2.895	1.984	1.456
	Age at diagnosis	−0.036	0.035	−0.163	0.307	−0.108	0.035	1.198
	Tumor size (mm)	0.004	0.025	0.027	0.891	−0.048	0.055	1.785
	Depth of invasion (mm)	0.038	0.041	0.166	0.364	−0.046	0.122	1.573
	Histology grade	−1.020	0.917	−0.185	0.274	−2.884	0.845	1.340
	Necrosis presence	0.510	1.300	0.062	0.697	−2.131	3.152	1.196
	Vascular invasion	−0.573	1.344	−0.067	0.673	−3.304	2.158	1.182
	Perineural invasion	−2.661	1.133	−0.382	0.025	−4.963	−0.359	1.282
	Extracapsular nodal extension	−0.183	2.271	−0.013	0.936	−4.798	4.432	1.303
	Stage	0.263	0.629	0.076	0.678	−1.015	1.541	1.598
	Smoking	3.056	1.546	0.319	0.056	−0.086	6.197	1.258

2	(Constant)	2.393	2.998		0.430	−3.693	8.480	
	Sex: male	−0.456	1.183	−0.066	0.703	−2.858	1.947	1.456
	Age at diagnosis	−0.036	0.034	−0.161	0.301	−0.105	0.034	1.174
	Tumor size (mm)	0.004	0.025	0.029	0.875	−0.046	0.054	1.725
	Depth of invasion (mm)	0.038	0.041	0.165	0.359	−0.045	0.120	1.571
	Histology grade	−1.040	0.869	−0.189	0.240	−2.805	0.725	1.238
	Necrosis presence	0.499	1.274	0.060	0.698	−2.087	3.085	1.182
	Vascular invasion	−0.558	1.313	−0.065	0.673	−3.224	2.107	1.161
	Perineural invasion	−2.669	1.113	−0.383	0.022	−4.928	−0.409	1.273
	Stage	0.254	0.609	0.073	0.679	−0.983	1.491	1.544
	Smoking	3.070	1.514	0.320	0.050	−0.003	6.143	1.241
6	(Constant)	2.699	2.666		0.318	−2.693	8.091	
	Age at diagnosis	−0.034	0.032	−0.150	0.306	−0.099	0.032	1.156
	Depth of invasion (mm)	0.048	0.032	0.211	0.139	−0.016	0.113	1.073
	Histology grade	−0.901	0.778	−0.163	0.254	−2.475	0.672	1.093
	Vascular invasion	−0.470	1.241	−0.055	0.707	−2.981	2.040	1.144
	Perineural invasion	−2.650	1.012	−0.381	0.013	−4.697	−0.603	1.161
	Smoking	2.804	1.314	0.292	0.039	0.146	5.461	1.031

7	(Constant)	2.845	2.609		0.282	−2.429	8.119	
	Age at diagnosis	−0.036	0.031	−0.162	0.253	−0.099	0.027	1.100
	Depth of invasion (mm)	0.048	0.032	0.211	0.134	−0.016	0.112	1.073
	Histology grade	−0.922	0.768	−0.167	0.237	−2.473	0.629	1.088
	Perineural invasion	−2.747	0.969	−0.394	0.007	−4.705	−0.788	1.087
	Smoking	2.825	1.299	0.295	0.036	0.201	5.450	1.029

B, regression coefficient; SE, standard error; beta, standard regression coefficient; CI, confidence interval; VIF, variance inflation factor.

## Data Availability

The data are available from the corresponding author upon reasonable request.

## References

[1] Menini M., de Giovanni E., Bagnasco F. (2021). Salivary micro-RNA and oral squamous cell carcinoma: a systematic review. *Journal of Personalized Medicine*.

[2] Starzyńska A., Sejda A., Adamska P. (2021). Prognostic value of the PIK3CA, AKT, and PTEN mutations in oral squamous cell carcinoma: literature review. *Archives of Medical Science*.

[3] Elahi M., Rakhshan V. (2020). MED15, transforming growth factor beta 1 (TGF-*β*1), Fc*γ*RIII (CD16), and HNK-1 (CD57) are prognostic biomarkers of oral squamous cell carcinoma. *Scientific Reports*.

[4] Yang L. (2010). TGF*β* and cancer metastasis: an inflammation link. *Cancer and Metastasis Reviews*.

[5] Tian M., Schiemann W. P. (2009). The TGF-*β* paradox in human cancer: an update. *Future Oncology*.

[6] Elahi M., Rakhshan V., Ghasemian N. T., Moshref M. (2012). Prognostic value of transforming growth factor beta 1 [TGF-*β*1] and matrix metalloproteinase 9 [MMP-9] in oral squamous cell carcinoma. *Biomarkers*.

[7] Taghavi N., Bagheri S., Akbarzadeh A. (2016). Prognostic implication of CD57, CD16, and TGF-beta expression in oral squamous cell carcinoma. *Journal of Oral Pathology & Medicine*.

[8] Topham N. J., Hewitt E. W. (2009). Natural killer cell cytotoxicity: how do they pull the trigger?. *Immunology*.

[9] Longworth M. S., Laimins L. A. (2004). The binding of histone deacetylases and the integrity of zinc finger-like motifs of the E7 protein are essential for the life cycle of human papillomavirus type 31. *Journal of Virology*.

[10] Fraga C. A. D. C., Oliveira M. V. M. D., Domingos P. L. B. (2012). Infiltrating CD57+ inflammatory cells in head and neck squamous cell carcinoma: clinicopathological analysis and prognostic significance. *Applied Immunohistochemistry & Molecular Morphology*.

[11] Wangerin H., Kristiansen G., Schlomm T. (2014). CD57 expression in incidental, clinically manifest, and metastatic carcinoma of the prostate. *BioMed Research International*.

[12] Nasir A., Catalano E., Calafati S., Cantor A., Kaiser H. E., Coppola D. (2004). Role of p53, CD44V6 and CD57 in differentiating between benign and malignant follicular neoplasms of the thyroid. *In Vivo*.

[13] Khan A., Baker S. P., Patwardhan N. A., Pullman J. M. (1998). CD57 (leu-7) expression is helpful in diagnosis of the follicular variant of papillary thyroid carcinoma. *Virchows Archiv*.

[14] Adler D., Offermann A., Halbach R. (2015). Clinical and molecular implications of MED15 in head and neck squamous cell carcinoma. *American Journal of Pathology*.

[15] Malik S., Roeder R. G. (2010). The metazoan mediator co-activator complex as an integrative hub for transcriptional regulation. *Nature Reviews Genetics*.

[16] Solana R., Mariani E. (2000). NK and NK/T cells in human senescence. *Vaccine*.

[17] Solana R., Pawelec G., Tarazona R. (2006). Aging and innate immunity. *Immunity*.

[18] Camous X., Pera A., Solana R., Larbi A. (2012). NK cells in healthy aging and age-associated diseases. *Journal of Biomedicine and Biotechnology*.

[19] Butcher S. K., Chahal H., Nayak L. (2001). Senescence in innate immune responses: reduced neutrophil phagocytic capacity and CD16 expression in elderly humans. *Journal of Leukocyte Biology*.

[20] Foster A. D., Sivarapatna A., Gress R. E. (2011). The aging immune system and its relationship with cancer. *Aging Health*.

[21] Tilden A. B., Grossi C. E., Itoh K., Cloud G. A., Dougherty P. A., Balch C. M. (1986). Subpopulation analysis of human granular lymphocytes: associations with age, gender and cytotoxic activity. *Natural Immunity and Cell Growth Regulation*.

[22] Bergmann S., Siekmeier R., Mix C., Jaross W. (1998). Even moderate cigarette smoking influences the pattern of circulating monocytes and the concentration of sICAM-1. *Respiration Physiology*.

[23] Moszczynski P., Zabinski Z., Moszczynski P., Rutowski J., Slowinski S., Tabarowski Z. (2001). Immunological findings in cigarette smokers. *Toxicology Letters*.

[24] Moustafa S. M., El-elemi A. H. (2013). Evaluation of probable specific immunotoxic effects of cigarette smoking in smokers. *Egyptian Journal of Food Science*.

[25] Hockertz S., Emmend-rffer A., Scherer G. (1994). Acute effects of smoking and high experimental exposure to environmental tobacco smoke (ETS) on the immune system. *Cell Biology and Toxicology*.

[26] Olloquequi J., Montes J. F., Prats A. (2011). Significant increase of CD57+ cells in pulmonary lymphoid follicles of COPD patients. *European Respiratory Journal*.

[27] Nakamura H., Ogawa Y., Nagase H. (2001). Natural killer cell activity and its related psychological factor, sense of coherence in male smokers. *Journal of Occupational Health*.

[28] Tanigawa T., Araki S., Nakata A. (1998). Increase in memory (CD4+CD29+ and CD4+CD45RO+) T and naive (CD4+CD45RA+) T-cell subpopulations in smokers. *Archives of Environmental Health: An International Journal*.

[29] Qiu F., Liang C.-L., Liu H. (2017). Impacts of cigarette smoking on immune responsiveness: up and down or upside down?. *Oncotarget*.

[30] Saetta M., Di Stefano A., Turato G. (1998). CD8+ T-lymphocytes in peripheral airways of smokers with chronic obstructive pulmonary disease. *American Journal of Respiratory and Critical Care Medicine*.

[31] Centaro E., Landini L., Leone A. (2012). Cigarette smoking and angiogenesis: what is the role of endothelial progenitor cells?. *Current Angiogenesis*.

[32] Feng A. L., Zhu J. K., Sun J. T. (2011). CD16+ monocytes in breast cancer patients: expanded by monocyte chemoattractant protein-1 and may be useful for early diagnosis. *Clinical and Experimental Immunology*.

[33] Ishigami S., Natsugoe S., Tokuda K. (2000). Clinical impact of intratumoral natural killer cell and dendritic cell infiltration in gastric cancer. *Cancer Letters*.

[34] Akagi J., Baba H. (2008). Prognostic value of CD57(+) T lymphocytes in the peripheral blood of patients with advanced gastric cancer. *International Journal of Clinical Oncology*.

[35] Fang J., Li X., Ma D. (2017). Prognostic significance of tumor infiltrating immune cells in oral squamous cell carcinoma. *BMC Cancer*.

[36] Lopes M. L. D. D. S., Barros C. C. D. S., Medeiros M. R. D. S. (2017). Evaluation of CD57+ cells in oral squamous cells carcinoma and their relationship with clinicopathological parameters. *JORDI - Journal of Oral Diagnosis*.

[37] Iida M., Takayama E., Naganawa K. (2014). Increase of peripheral blood CD57+ T-cells in patients with oral squamous cell carcinoma. *Anticancer Research*.

[38] Samanta D., Gonzalez A. L., Nagathihalli N., Ye F., Carbone D. P., Datta P. K. (2012). Smoking attenuates transforming growth factor-*β*-mediated tumor suppression function through down-regulation of smad3 in lung cancer. *Cancer Prevention Research*.

[39] Zhang Y., Pan T., Zhong X., Cheng C. (2014). Nicotine upregulates microRNA-21 and promotes TGF-beta-dependent epithelial-mesenchymal transition of esophageal cancer cells. *Tumor Biology*.

[40] Logullo A. F., Nonogaki S., Miguel R. E. (2003). Transforming growth factor *β*1 (TGF*β*1) expression in head and neck squamous cell carcinoma patients as related to prognosis. *Journal of Oral Pathology & Medicine*.

